# Infective Endocarditis and Excessive Use of B− Blood Type Due to Surgical Treatment—Is It Only a Local Problem? LODZ-ENDO Results (2015–2025)

**DOI:** 10.3390/jcm14228101

**Published:** 2025-11-15

**Authors:** Robert Morawiec, Karolina Mlynczyk, Michal Krejca, Jaroslaw Drozdz

**Affiliations:** 1 2nd Department of Cardiology, Medical University of Lodz, Pomorska 251, 92-213 Lodz, Poland; 2 Laboratory of Transfusion Immunology with the Blood Bank MLD-CKD of Lodz, Pomorska 251, 92-213 Lodz, Poland; 3 Department of Cardiac Surgery, Medical University of Lodz, Pomorska 251, 92-213 Lodz, Poland

**Keywords:** endocarditis, bacterial, blood group antigens, transfusion medicine

## Abstract

**Background/Objectives**: Infective endocarditis (IE) remains a rare but increasingly complex condition, posing significant challenges for cardiologists and cardiac surgeons. Blood groups from the ABO/Rh system have been associated with susceptibility to various diseases, including infections and bacterial colonization tendencies. However, data on the distribution of ABO/Rh blood types among IE patients are lacking. We hypothesized that the prevalence of ABO/Rh blood types among IE patients differs from their frequency in the general population. This study aimed to assess the distribution of ABO/Rh blood types in the LODZ-ENDO database in comparison to general populations. **Methods**: LODZ-ENDO is a single-center retrospective study conducted in a tertiary cardiology and cardiac surgery facility serving 2.35 million residents. All consecutive patients with confirmed IE hospitalized between 1 January 2015 and 1 October 2025 were included. Clinical data and ABO/Rh blood types were collected and compared with national population data using Fisher’s exact and chi-square tests. **Results:** A total of 329 patients with IE were analyzed (median age 61 (41–68) years; 69% men), of whom 227 underwent cardiac surgery. Overall ABO/Rh distribution differed significantly from the general population (*p* = 0.033), driven by a tendency to an overrepresentation of B− (LODZ-ENDO 5.2% vs. Poland 2%; OR 2.88; 95% CI 1.17–7.29; *p* = 0.03; power 0.89; *p*(adj) = 0.23). Considering regional demographics and blood use (≈3 units per surgery), this represents an excess annual use of 1.9 B− units, equal to 0.23% of regional B− reserves, with additional indirect depletion of O− blood. Based on WHO data, if this overrepresentation exists elsewhere, IE-related surgeries could consume 0.2–1.3% of national B− stocks in smaller European countries such as Malta, Iceland, Luxembourg, Cyprus, Estonia, Lithuania, Latvia, and Slovenia. **Conclusions:** This, probably the first report of B− blood type overrepresentation in IE indicates disproportionate use of a rare blood group, highlighting the need for targeted blood management strategies, especially near specialized cardiac surgery centers.

## 1. Background

Infective endocarditis (IE), though rare, has shown an increasing number of cases and growing clinical complexity over the last decades, becoming a significant challenge for cardiologists and cardiac surgeons [1]. Large national and European IE registries, such as POL-ENDO [2] and EURO-ENDO [3], provide valuable information on clinical presentations, complications, optimal management strategies, and mortality. However, some data remain limited.

One of the gaps in current evidence concerns the distribution of blood types (ABO/Rh) among patients with IE. We found no reports addressing this topic in medical publication databases. Different blood types within the ABO/Rh blood group system have been shown to be associated with varying susceptibility to pathological states and infections, as well as bacterial colonization tendencies [4]. For example, it has been demonstrated that pediatric patients with blood groups B and AB (*n* = 194) had a 55% higher risk of *E. coli* infection (*p* = 0.009) and a 131% higher risk of *Salmonella* infection (*p* = 0.007) [5]. In a large study involving 23,253 patients, blood groups B and AB were associated with a 60% increased risk of *E. coli* sepsis (RR 1.6, *p* = 0.01) [6].

## 2. Aim

Based on these observations, we hypothesized that the prevalence of ABO/Rh blood types among IE patients differs from their frequency in the general population. This study aimed to analyze the distribution and characteristics of ABO/Rh blood types in the LODZ-ENDO database and compare them with national population data.

## 3. Methods

LODZ-ENDO is a single-center, retrospective study conducted at a tertiary cardiology and cardiac surgery facility serving approximately 2.35 million residents. All consecutive patients with confirmed IE hospitalized between 1 January 2015 and 1 October 2025 were included. The diagnosis of IE was established according to Duke’s criteria and/or confirmed by transesophageal echocardiography. The study included patients with valve-related IE (VR-IE), while all individuals with any type of cardiac device-related infective endocarditis (CDRIE) were excluded. Each patient received guideline-directed antimicrobial treatment. All patients in the LODZ-ENDO database were referred to our tertiary center with standard indications for cardiac surgery according to the ESC guidelines. For each patient, a final evaluation was performed by an experienced Heart Team to determine whether surgical or conservative management was most appropriate. We analyzed demographic data (sex, age), management strategy (operative or conservative), echocardiographic findings (IE localization, number of valves affected, left ventricular ejection fraction [LVEF]), basic comorbidities (presence of diabetes mellitus and chronic obstructive pulmonary disease), blood culture results, and basic laboratory findings from blood samples collected at admission (including complete blood count, C-reactive protein [CRP], N-terminal prohormone of brain natriuretic peptide [NT-proBNP], and estimated glomerular filtration rate [eGFR]).

Among all ABO/Rh blood types, we analyzed sex, age, management strategy (conservative vs. surgical treatment), and in-hospital mortality in VR-IE patients. Data on ABO/Rh blood types were collected and compared with the national population. Information on specific blood type frequencies and blood donations was obtained from the National Blood Center (Polish: *Narodowe Centrum Krwi*) [7], the official blood donors’ website [8], and Statistics Poland (Polish: *Główny Urząd Statystyczny*) [9] (Table 1). Data on blood donations across Europe (adjusted for national income levels) were obtained from the World Health Organization (WHO) [10] and publicly available national blood center databases.

The LODZ-ENDO study was reviewed by the Bioethical Committee of the Medical University of Lodz (opinion number: RNN/135/25/KE, 15 April 2025).

### Statically Analysis

Statistical analyses were performed using STATA software (version 17.0; StataCorp LLC, College Station, TX, USA). Continuous variables are presented as means with standard deviations (SDs) or medians with interquartile ranges (IQRs), depending on data distribution, which was verified using the Shapiro–Wilk test.

Comparisons between LODZ-ENDO data and population blood type frequencies were conducted using Fisher’s exact test and chi-square statistics. Comparisons among variables were performed using the chi-squared test or Kruskal–Wallis test (non-parametric one-way ANOVA on ranks), as appropriate. Significant results in multiple comparisons were calculated with Bonferroni correction. *p*-values < 0.05 were considered statistically significant.

## 4. Results

This analysis included 333 patients with confirmed VR-IE hospitalized between 1 January 2015 and 1 October 2025. Data on blood type were missing for 4 subjects; thus, the final analysis included 329 patients. Among them, 227 underwent cardiac surgery. Most patients were men, with a median age of 61 years [IQR: 41–68 years], left-sided IE (mitral or aortic valve), and the overall in-hospital mortality rate was 29.7% (Table 2). The analyzed population presented with elevated median NT-proBNP and CRP levels at admission and preserved median LVEF. Among patients with an identified etiological factor for IE, the most common cause was *Staphylococcus* spp., followed by *Enterococcus* spp. and *Streptococcus* spp. In almost half of the cases, the etiological factor remained unidentified (Table 2). The results of the ABO/Rh analysis are presented in Table 2. No statistically significant differences were observed in sex, age, or mortality across blood type subgroups. The longest median time from admission to surgery, although not statistically significant, was observed in patients with blood types O and B+/B−.

The overall ABO/Rh distribution differed from that of the general population (χ^2^ = 14.3, *p* = 0.033) (Table 3). Although the B− blood group appeared overrepresented—5.2% in LODZ-ENDO vs. 2% in the general population (OR = 2.88, 95% CI [1.17–7.29]; *p* = 0.03), this association did not remain statistically significant after Bonferroni correction for multiple comparisons (adjusted *p* = 0.23).

**Figure 1:**
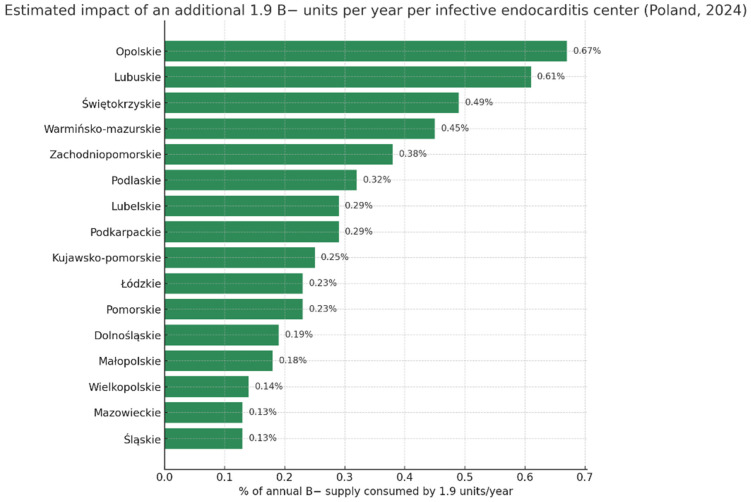
Estimated impact of additional 1.9 B− units per year per one infective endocarditis surgical treatment center (by voivodeship/province, Poland 2024).

Considering the number of patients requiring cardiac surgery (227 between 2015 and 2025), regional demographics (2,355,000 residents), the number of donors per 10,000 citizens (172.8 in 2024 [9]), and expected B− donations based on national distribution [7], we estimated that this represents an excess annual use of 1.9 B− units—equivalent to 0.23% of regional and 0.015% of national B− reserves, from only one site treating IE surgically. Depending on the voivodeship (province) in Poland, this annual excess use by a single center may reach up to 0.67% of regional B− reserves (Table 4, Figure 1).

**Figure 2:**
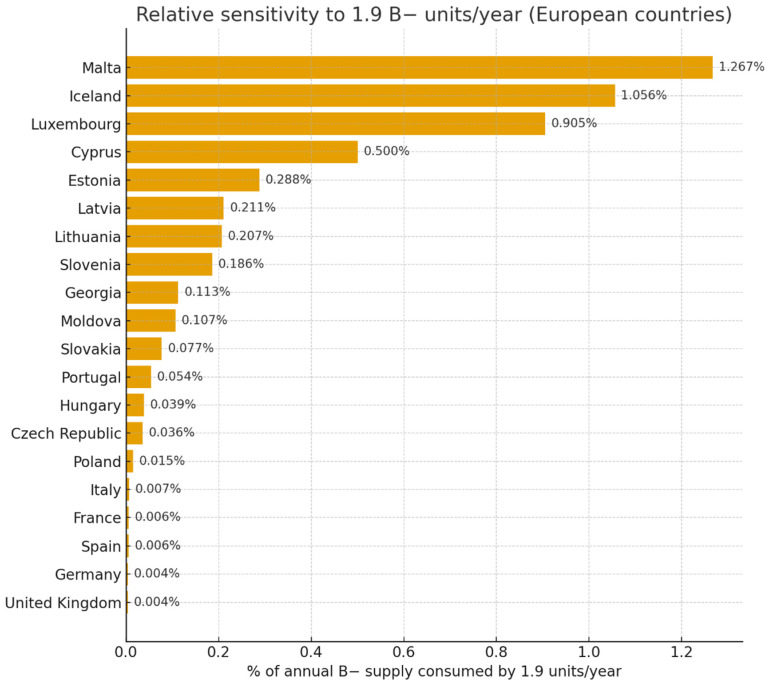
Potential significance of an additional 1.9 B− units per year per one infective endocarditis surgical treatment center (European countries, 2024–2025).

Furthermore, considering that B− overrepresentation may be typical among IE patients, analysis of online data from European national blood centers suggests that in smaller European countries, this excess annual use per surgical center could reach 0.2–1.3% of national B− reserves (e.g., Malta, Iceland, Luxembourg, Cyprus, Estonia, Lithuania, Latvia, and Slovenia) (Table 5, Figure 2).

## 5. Discussion

To our knowledge, this is the first report analyzing the distribution of ABO/Rh blood types among patients with infective endocarditis. The main finding is a significant difference between IE patients and the general Polish population, characterized by a tendency to overrepresentation of individuals with the B− blood type. Although no associations were observed between blood type and age, sex, or in-hospital mortality, this finding suggests a potential biological or epidemiological link between blood group antigens and susceptibility to IE.

In our region, this overrepresentation leads to an additional annual use of approximately 1.9 units of B− blood. Given that B− is a rare blood type—occurring in about 2% of the Polish population and 1.1–3% in Europe (mean ~2%) [11]—even minor increases in demand may have a tangible impact on regional blood supplies. Because B− patients can only receive B− or universal O− blood, excessive use of B− may indirectly strain O− reserves as well. Consequently, each IE cardiac surgery center may significantly affect regional blood management, as supplies of B− and O− blood must also cover other clinical indications.

Naturally occurring ABO antibodies (anti-A in individuals with blood groups B and AB and anti-B in those with blood groups A and AB) are involved in the innate immune defense against bacterial pathogens [4]. This phenomenon was demonstrated in a study by Check et al., in which human polyclonal anti-B antibodies, but not anti-A, induced approximately a tenfold increase in neutrophil phagocytic activity (10.9 vs. 1.19 bacteria per cell) [5]. This observation could partly explain the higher representation of blood group B in our cohort, given that individuals with this blood type lack anti-B antibodies. Previous studies [12] have demonstrated that expression of the B antigen (in blood groups B and AB) is associated with distinct gut microbiota profiles compared with non-B antigen groups (A and O), including significant differences in the abundance and diversity of several bacterial taxa. Notably, blood groups B and AB exhibited the greatest microbial diversity, whereas group O showed the lowest. The higher microbial diversity observed in individuals with blood group B may partly reflect differences in host–microbe interactions and could be related to variations in innate immune defense mechanisms. These findings suggest that mucosal expression of the B antigen may influence gut microbiota composition and host immune responses, which could, in part, contribute to the higher prevalence of blood group B observed in our cohort.

Similarly to the ABO system, data regarding the role of Rh blood type in the host response to infection are inconsistent. Some studies have reported increased viral resistance in RhD-negative individuals, particularly against influenza A virus (potentially due to an amplified interferon-gamma signaling axis) [13] and COVID-19 [14]. Conversely, a meta-analysis by Butler et al. demonstrated an association between Rh-positive blood status and a higher risk of SARS-CoV-2 infection [15]. Some studies have suggested that Rh-positive status may be associated with a lower risk or milder course of *Babesia microti* infection [16] and possibly malaria [17]; however, evidence remains limited and inconclusive. Both of these are intraerythrocytic parasitic infections, which may partly explain the proposed association. Unlike the ABO system, which has been linked to various infectious and inflammatory processes, the Rh system shows very limited and inconsistent evidence for any association with bacterial sepsis. Rh antigens, on the other hand, are restricted to red blood cell membranes and not expressed on other tissues, which makes a direct immunological role in bacterial infection far less likely.

As mentioned, previous studies have demonstrated that ABO and Rh blood groups can influence susceptibility to various infectious diseases [4,12,13,14,15,16,17,18]. Blood group antigens may act as microbial receptors or modulate immune responses, affecting host susceptibility to infection. In particular, blood groups B and AB have been associated with higher risks of *E. coli* and *Salmonella* infections [5,6] and altered bacterial adhesion profiles. Atefi et al. reported a significant difference in ABO blood type prevalence compared with the general population (*p* = 0.036), but no difference in the Rh system [19]. In another study involving 6296 patients, the authors found a higher risk of sepsis-associated thrombocytopenia in blood groups B (OR = 1.32; 95% CI: 1.05–1.67) and O (OR = 1.37; 95% CI: 1.09–1.72) compared with blood group AB [20]. The tendency to overrepresentation of B− in our cohort may therefore reflect differences in pathogen–host interactions, immune mechanisms, or bacterial colonization patterns that predispose certain individuals to endocardial infection.

We also identified a small study (*n* = 60) showing that individuals with the B− blood type had significantly higher epidemiological indices of dental caries (Decayed, Missing, and Filled Teeth—DMFT) [21]. Another study of 100 participants found the highest mean DMFT in those with blood group B, although this result was not statistically significant [22]. Gautam et al. found a greater prevalence of periodontitis in blood group B among 537 subjects and higher gingivitis and periodontitis among Rhesus positive groups [23]. Given that dental caries is one of the most common causes of IE [1], this may partially explain the observed overrepresentation of B blood type in our cohort, though data remain limited.

The rarity of the B− blood group presents an additional challenge for transfusion services. Even a small increase in the number of surgical IE patients with this type can have measurable consequences for blood reserves. In our analysis, the excess annual B− use from one surgical center corresponded to 0.23% of regional and 0.015% of national B− reserves. While these percentages appear modest, they represent a real burden on already limited stocks—especially given that B− recipients can receive blood only from B− or O− donors. This situation indirectly increases pressure on O− reserves, crucial for emergency transfusions. These findings highlight the interplay between clinical epidemiology and transfusion management, emphasizing the need for proactive blood demand forecasting in cardiac surgery centers, especially given the documented rise in IE incidence over recent decades [24].

From a clinical standpoint, the relationship between blood type and IE risk remains speculative. However, if confirmed in larger multicenter studies, this association could enhance understanding of host-related risk factors, support more accurate epidemiological modeling of IE incidence, and improve resource planning. Our findings underscore that even small demographic shifts in patient populations can have disproportionate logistical consequences, particularly regarding blood resource allocation.

## 6. Limitations

The strengths of this study include a clearly defined cohort of valve-related IE patients and a long observation period (2015–2025). However, several limitations should be noted. This was a single-center, retrospective study with a moderate but representative sample size (*n* = 329), which limits generalizability. Comparisons with national data relied on donor-based statistics that may not perfectly reflect the general population, as the distribution of blood groups among donors may differ slightly from the general population. Nevertheless, if confirmed by other studies, these findings may have important implications not only for clinical IE management but also for healthcare system planning, particularly in blood resource management.

Extrapolating single-center results to national populations may represent overfitting, as generalizability is limited. Therefore, the observed overrepresentation of B− should be interpreted cautiously and verified in larger, multicenter registries. However, our intention is to highlight the potential scale of this problem, which may extend beyond our region. Also, due to limited microbiological data (a high proportion of unknown or negative blood culture results [25]), we could not analyze associations between specific pathogens and blood types.

Future research should aim to confirm these findings in larger, prospective, multi-institutional studies. Integrating clinical and transfusion databases could provide more robust evidence on the relationship between blood group distribution and transfusion demand in IE. Mechanistic studies exploring pathogen–blood group interactions may help elucidate underlying biological pathways. Finally, healthcare policy frameworks should consider incorporating blood-type-based risk monitoring into regional blood management and planning systems.

## 7. Conclusions

This study demonstrates that the distribution of ABO/Rh blood groups among patients with infective endocarditis differs significantly from that in the general population, with an overrepresentation of the B− type. Although the biological basis of this association remains unclear, the implications for transfusion medicine and resource planning are evident. These findings emphasize the need for continued collaboration between clinical, epidemiological, and transfusion medicine disciplines to optimize care for patients with IE and ensure sustainable blood supply management.

## Figures and Tables

**Table 1:** Population data on AB0/RH blood types in Poland.

Blood Type	Frequency
A+	32%
A−	6%
B+	15%
B−	2%
AB+	7%
AB−	1%
0+	31%
0−	6%

**Table 2:** Baseline characteristic of study population (overall and among ABO/RH blood types).

Overall (*n* = 333)								
Women [*n*, (%)]			254 (77.2%)					
Men [*n*, (%)]			75 (22.8%)					
Age [Me, (IQR)]			61 (47–68)					
Left ventricle ejection fraction (%) [Me, (IQR)]			55 (48–60)					
Treatment—cardiac surgery [*n*, (%)]			227 (68.2%)					
In-hospital mortality			99 (30.1%)					
Diabetes mellitus			83 (24.9%)					
Chronic obstructive pulmonary disease			22 (6.6%)					
Left-sided IE			305 (91.6%)					
Right-sided IE			20 (6%)					
Both-sided IE			8 (2.4%)					
*Staphylococcus* spp.			81 (24.3%)					
*Enterococcus* spp.			38 (11.4%)					
*Streptococcus* spp.			28 (8.4%)					
Other			21 (6.3%)					
Unidentified			165 (49.5%)					
WBC (×10^6^/L) [Me, (IQR)]			9.3 (7.13–11.9)					
Lym (×10^6^/L) [Me, (IQR)]			1.41 (1.04–1.91)					
Neu (×10^6^/L) [Me, (IQR)]			6.83 (4.76–9.18)					
Plt (×10^6^/L) [Me, (IQR)]			239 (170–303)					
Hgb (g/dL) [Me, (IQR)]			10.5 (9.4–11.8)					
CRP (mg/L) [Me, (IQR)]			48.05 (22–101.9)					
NT-proBNP (pg/mL) [Me, (IQR)]			4859 (1785–18,575.5)					
eGFR (ml/min/1.73 m^2^) [Me, (IQR)]			66.6 (40.1–91.8)					
Blood types AB0/Rh [*n*, (%)] (*n* = 329)								
A+			103 (31.3%)					
A−			16 (4.9%)					
B+			52 (15.8%)					
B−			17 (5.2%)					
AB+			18 (5.5%)					
AB−			4 (1.2%)					
0+			100 (30.4%)					
0−			19 (5.8%)					
Blood types AB0/Rh (by sex) [*n*, (%)] (*n* = 329)								
			Men			Women		
A+			80 (31.5%)			23 (30.7%)		*p* = 0.36
A−			10 (3.9%)			6 (8%)		
B+			42 (16.5%)			10 (13.3%)		
B−			10 (3.9%)			7 (9.3%)		
AB+			15 (5.9%)			3 (4%)		
AB−			3 (1.2%)			1 (1.3%)		
0+			81 (31.9%)			19 (25.3%)		
0−			13 (5.1%)			6 (8%)		
Blood types AB0/Rh (age—median) (*n* = 329)								
A+			61			*p* = 0.21		
A−			61					
B+			55					
B−			65					
AB+			65					
AB−			62.5					
0+			58					
0−			63					
Blood types AB0/Rh—in-hospital mortality [*n*, (%)] (*n* = 329)								
A+			33 (33.3%)			*p* = 0.67		
A−			2 (2.0%)					
B+			18 (18.2%)					
B−			5 (5.1%)					
AB+			4 (4.0%)					
AB−			2 (2.0%)					
0+			28 (28.3%)					
0−			7 (7.1%)					
Time admission to surgery (days) [Me, (IQR)] (*n* = 227)								
A+			11 (7–21)			*p* = 0.82		
A−			9 (6.5–13)					
B+			13 (8–16)					
B−			13 (8.5–17.5)					
AB+			12.5 (7–14)					
AB−			- (*n* = 0)					
0+			11 (6–16)					
0−			15 (7–20)					
Blood types AB0/Rh by etiologic factor [*n*, (%)] (*n* = 329; % within each blood type] *p* = NS								
	A+	A−	B+	B−	AB+	AB−	0+	0−
*Staphylococcus* spp.	24 (23.3%)	1 (6.3%)	11 (21.2%)	6 (35.3%)	8 (44.4%)	4 (100%)	21 (21.0%)	5 (26.3%)
*Enterococcus* spp.	11 (10.7%)	1 (6.3%)	8 15.4%)	2 (11.8%)	3 (16.7%)	0	10 (10.0%)	3 (15.8%)
*Streptococcus* spp.	8 (7.8%)	2 (12.5%)	7 (13.5%)	0	1 (5.6%)	0	10 (10.0%)	0
Other	10 (9.7%)	1 (6.3%)	2 (3.8%)	0	0	0	4 (4.0%)	4 (21.1%)
Unidentified	50 (48.5%)	11 (68.8%)	24 (46.2%)	9 (52.9%)	6 (33.3%)	0	55 (55.0%)	7 (36.8%)

WBC—white blood count; Lym—Lymphocytes count; Plt—Platelet count; Neu—Neutrophils count; Hgb—Hemoglobin; CRP—C-reactive protein; NT-proBNP—N-terminal prohormone of brain natriuretic peptide; eGFR—Estimated Glomerular Filtration Rate.

**Table 3:** Blood types (Ab0/Rh) distribution—LODZ-ENDO vs. Polish population (*p* = 0.033).

Blood Type (AB0/Rh)	Number Observed	Number Expected	% Observed	% Expected	OR	95% CI OR	*p* (Fisher)	*p* (χ^2^)	Test Power
A+	105	105.3	31.9	32	1.0	[0.72–1.39]	0.93	0.96	0.06
A−	16	19.7	4.9	6	0.81	[0.43–1.52]	0.6	0.69	0.3
B+	52	49.4	15.8	15	1.06	[0.71–1.58]	0.81	0.82	0.05
B−	17	6.6	5.2	2	2.88	[1.17–7.29]	0.029/0.232 *	0.031/0.248 *	0.89
AB+	18	23	5.5	7	0.77	[0.42–1.41]	0.41	0.49	0.17
AB−	4	3.3	1.2	1	1.21	[0.33–4.40]	0.73	0.75	0.05
0+	98	102	29.8	31	0.96	[0.69–1.34]	0.81	0.83	0.07
0−	19	19.7	5.8	6	0.97	[0.54–1.75]	1	1	0.05
Rh^+^ (total)	252	279	83.0	85.0	0.83	[0.53–1.28]	0.49	0.48	0.19
Rh^−^ (total)	52	50	17.0	15.0	1.17	[0.76–1.80]	0.49	0.48	0.19
A (total)	114	125	34.7	38.0	0.86	[0.65–1.13]	0.30	0.27	0.25
B (total)	62	56	18.9	17.0	1.13	[0.80–1.60]	0.51	0.48	0.23
AB (total)	20	26	6.1	8.0	0.74	[0.44–1.24]	0.29	0.27	0.27
O (total)	109	111	33.1	33.0	1.00	[0.75–1.33]	0.99	0.98	0.05
Overall AB0/Rh	-	-	-	-	-	-	-	0.03 **	-

* after Bonferroni correction, ** global χ^2^ test for entire 8 × 2 distribution (ABO × Rh).

**Table 4:** Estimated impact of additional 1.9 B− units per year per one infective endocarditis surgical treatment center (by voivodeship/province, Poland 2024).

Region	Population	Donors Per 10,000 [9]	Annual Donations	Estimated B− Supply (2%)	% of B− Supply Consumed by 1.9 Units/Site/Year
POLAND (total)	38,290,000	172.5	617,000	12,340	0.015%
Dolnośląskie	2,875,000	171.1	49,200	984	0.193%
Kujawsko-Pomorskie	1,991,000	193.3	38,500	770	0.247%
Lubelskie	2,005,000	163.6	32,800	656	0.290%
Lubuskie	975,000	160.0	15,600	312	0.609%
Łódzkie	2,355,000	172.8	40,700	814	0.233%
Małopolskie	3,427,000	154.9	53,100	1062	0.179%
Mazowieckie	5,512,000	132.4	73,000	1460	0.130%
Opolskie	933,000	152.2	14,200	284	0.669%
Podkarpackie	2,066,000	157.8	32,600	652	0.291%
Podlaskie	1,134,000	262.8	29,800	596	0.319%
Pomorskie	2,361,000	173.7	41,000	820	0.232%
Śląskie	4,304,000	166.1	71,500	1430	0.133%
Świętokrzyskie	1,164,000	167.6	19,500	390	0.487%
Warmińsko-Mazurskie	1,352,000	157.5	21,300	426	0.446%
Wielkopolskie	3,484,000	200.4	69,800	1396	0.136%
Zachodniopomorskie	1,627,000	153.7	25,000	500	0.380%

**Table 5:** Potential significance of an additional 1.9 B− units per year per one infective endocarditis surgical treatment center (European countries, 2024–2025).

Country	Population (Million)	World Bank Income	Donations/1000	Annual Donations	B− (%)	Annual B− Supply	% of B− Supply Consumed by 1.9 Units/Site/Year
Poland	38.3	Upper-middle	16.4	624,000	2.0	12,480	0.015%
Germany	84.1	High	31.5	2,647,000	2.0	52,940	0.004%
France	68.4	High	31.5	2,155,000	1.4	30,170	0.006%
Spain	47.3	High	31.5	1,490,000	2.0	29,800	0.006%
Italy	61.0	High	31.5	1,922,000	1.5	28,830	0.007%
United Kingdom	68.5	High	31.5	2,159,000	2.0	43,180	0.004%
Portugal	10.2	High	31.5	321,000	1.1	3530	0.054%
Czech Republic	10.8	Upper-middle	16.4	177,000	3.0	5310	0.036%
Hungary	9.9	Upper-middle	16.4	162,000	3.0	4860	0.039%
Slovakia	5.6	Upper-middle	16.4	92,000	2.7	2480	0.077%
Slovenia	2.1	Upper-middle	16.4	34,000	3.0	1020	0.186%
Estonia	1.34	Upper-middle	16.4	22,000	3.0	660	0.288%
Latvia	1.85	Upper-middle	16.4	30,000	3.0	900	0.211%
Lithuania	2.8	Upper-middle	16.4	46,000	2.0	920	0.207%
Iceland	0.36	High	31.5	11,000	1.6	180	1.056%
Malta	0.47	High	31.5	15 000	1.0	150	1.267%
Luxembourg	0.67	High	31.5	21,000	1.0	210	0.905%
Cyprus	1.32	High	31.5	42,000	0.9	380	0.500%
Moldova	3.6	Upper-middle	16.4	59,000	3.0	1770	0.107%
Georgia	4.9	Upper-middle	16.4	80,000	2.1	1680	0.113%

## Data Availability

The data presented in this study are available on request from the corresponding author.
